# Long-term follow-up of patients with pulmonary hypertension after catheter ablation of atrial fibrillation and atrial tachycardia

**DOI:** 10.3389/fcvm.2026.1826752

**Published:** 2026-06-18

**Authors:** Milan Dusik, Stepan Havranek, Zdenka Fingrova, Pavel Jansa, David Ambroz, Vladimir Dytrych, Tomas Skala, Martin Hutyra, Jan Precek, Adrian Reichenbach, Dan Wichterle

**Affiliations:** 1 2nd Department of Medicine—Department of Cardiovascular Medicine of the 1st Faculty of Medicine and General University Hospital in Prague, Prague, Czechia; 2 First Department of Internal Medicine—Cardiology, Olomouc University Hospital, Olomouc, Czechia; 3 Department of Cardiology, Institute of Clinical and Experimental Medicine, Prague, Czechia

**Keywords:** arrhythmia, atrial fibrillation, atrial tachycardia, catheter ablation, long-term follow-up, pulmonary hypertension

## Abstract

**Introduction:**

The previously published randomized trial comparing an extensive (targeting both clinical arrhythmia and bi-atrial arrhythmogenic substrate) and a limited (clinical arrhythmia only) approach to catheter ablation in patients with pulmonary hypertension did not demonstrate a significant difference in atrial tachyarrhythmia (ATA) recurrence during the one-year follow-up. This study presents the long-term results of these patients.

**Methods:**

Participants enrolled in the randomized trial (NCT04053361) were further followed and checked for ATA recurrence and all-cause mortality. Follow-up was primarily clinically driven, as the protocol for this secondary analysis was not fully prespecified

**Results:**

A total of 74 patients [41 males, median age 71 years (IQR: 61; 75)] were randomized to the extended (*N* = 36) and limited (*N* = 38) ablation groups and followed for a median of 45 and 41 months, respectively. The ATAs recurred in 22 (61%) patients after extended vs. 19 (50%) patients after limited ablation [hazard ratio (HR) 1.22, 95% confidence interval (CI): 0.66–2.3]. There were 21 (58%) deaths in the extended ablation group, and 22 (58%) patients died during follow-up after the limited ablation (HR: 0.74, 95% CI: 0.39–1.4). No recurrence of typical atrial flutter was noted among the 23 patients treated by the ablation of the cavo-tricuspid isthmus.

**Conclusion:**

Extensive catheter ablation in patients with pulmonary hypertension did not reduce ATA recurrence during the routine follow-up of more than 40 months. Cavo-tricuspid isthmus ablation appears effective in preventing recurrence of typical atrial flutter, even in this high-risk population. Given the exploratory nature of these analyses, further research is needed.

## 1 Introduction

Pulmonary hypertension (PH) is defined by the presence of elevated pulmonary arterial pressure. Previously, PH was diagnosed in patients with a pulmonary arterial mean pressure (PAMP) of 25 mmHg or more (1). Later, the upper limit of the normal PAMP was lowered to 20 mmHg (2). The worldwide prevalence of PH is estimated to be around 1% and is generally associated with greater morbidity and mortality (3).

Atrial tachyarrhythmias (ATAs), including atrial fibrillation and typical atrial flutter, are common in patients with PH. The prevalence of ATAs in all types of PH is 26%–31% (4). The presence of ATA is often connected with further clinical deterioration in those patients, and sinus rhythm restoration can relieve a patient's symptoms (5–8). Despite the high prevalence and clinical significance, the specific arrhythmogenic mechanisms as well as the optimal treatment strategy for heart rhythm disorders in PH patients are poorly understood (9).

Catheter ablation (CA) is a generally safe and effective method for rhythm control in patients with atrial fibrillation and other atrial tachycardias. According to the available data, it can be performed even in the presence of PH (10). However, the optimal approach to CA in this population has not yet been established, and the ATA recurrence despite ablation is common (10–12). Moreover, the subsequent occurrence of new and different types of ATAs further complicates sinus rhythm maintenance in those patients (11, 12).

In 2023, our group published the results of a multicenter randomized trial comparing CA limited to clinical ATA with the extended approach targeting potential arrhythmogenic substrate in both atria of patients with PH. Surprisingly, extensive ablation did not reduce the ATA recurrence rate during the follow-up period with a median duration of 13–14 months (13).

Follow-up of the patients originally randomized in this study continued, and we now present the long-term results comparing the limited and extended ablation strategies of CA in patients with PH.

## 2 Methods

We analyzed the long-term follow-up of patients originally included in the multicenter randomized trial named “Catheter ablation of atrial fibrillation and atrial tachycardia in patients with pulmonary hypertension: a randomized study” and registered at ClinicalTrials.gov under NCT04053361.

Recruitment for the study took place from May 2018 to August 2021. Further details and the complete results of the trial have already been published elsewhere (13). In brief, adults with pre-capillary or combined post- and pre-capillary PH of any etiology and documented symptomatic atrial fibrillation or atrial tachycardia (typical atrial flutter included) indicated for CA using radiofrequency energy were randomized 1:1 into two parallel treatment arms. The “limited ablation group” underwent ablation of clinical ATA only. The ablation in the “extended ablation group” targeted both clinical ATA and further arrhythmogenic substrate present in both atria. This consisted of an empirical lesion set within the right atrium: superior vena cava isolation, posteroseptal intercaval line, and cavo-tricuspid isthmus ablation and homogenization of low-voltage zones in both left and right atria (bipolar voltage <0.5 mV in sinus rhythm or <0.2 mV in ATA). The primary endpoint of the study was documented ATA recurrence lasting more than 30 s in patients without antiarrhythmic drugs. The basic follow-up was planned for 12 months after the CA at regular 3-month intervals, with a 3-month blanking period after the index ablation (13).

The secondary analysis of long-term outcomes was planned, but no further details were prespecified in the original study protocol. The follow-up included all participants initially randomized in the original trial. Study visits, including symptom assessment, physical examination, and ECG recording, were scheduled regularly at each study centre according to patients' clinical status. Additional telephone visits were performed when necessary. The arrhythmia events were collected from hospital and outpatient records. Dates of all-cause death were extracted from the Czech Central Healthcare Insurance Database.

All centers participating in the long-term follow-up were tertiary care hospitals and established expert centers for the treatment of pulmonary hypertension. Therefore, substantial inter-center differences in follow-up procedures, diagnostic methods, and treatment strategies were considered unlikely. All patients were managed in accordance with current clinical guidelines. The use of antiarrhythmic drugs was left to the discretion of the treating physician. In the event of ATA recurrence, electrical or pharmacological cardioversion was considered, and all patients were evaluated for repeat ablation. Patients in both study groups received identical management during follow-up, regardless of their original randomization assignment.

The primary endpoint of the long-term follow-up was documented ATA recurrence lasting more than 30 s. The secondary outcomes were all-cause mortality, time to ATA recurrence, and time to death. The analysis adhered to the original randomized allocation to the study groups.

The study was approved by the multicentre and local Ethics committees at all centres. Individual written consent was obtained from each patient. This analysis was performed in accordance with good clinical practice and the Helsinki Declaration.

### 2.1 Statistical analysis

All study objectives were analyzed by standard statistical methods (*t*-test or Mann–Whitney *U*-test for continuous variables, or a Chi-square or two-tailed Fisher exact test for categorical variables). Time-to-event data were analyzed using the Kaplan–Meier method with log-rank statistics and Cox proportional-hazards regression. A *P*-value < 0.05 was considered significant. All analyses were performed using the STATISTICA vers. 12 software (StatSoft, Inc., Tulsa, USA). No imputation methods for missing data were used.

## 3 Results

A total of 77 patients were randomized across 3 study centres in the Czech Republic. Subsequently, left atrial appendage thrombosis was detected in one patient in the limited ablation group, while severe mitral regurgitation was diagnosed in one patient in the extended ablation group. Another patient in the extended ablation group declined participation. None of these three patients underwent catheter ablation and were therefore excluded from the original trial., leaving 74 patients [41 males, median age 71 years (IQR: 61; 75)] for further analysis. The limited ablation group consisted of 38 subjects, and there were 36 subjects in the extended ablation group. The patients' baseline characteristics are summarized in Table 1.

**Table 1:** Baseline characteristics of the study groups.

	**All patients**	**Limited ablation**	**Extended ablation**	***P***
	***N* = 74**	***N* = 38**	***N* = 36**	
Age (years)	71 (61; 75)	70 (61; 75)	71 (60; 74)	0.61
Males	41 (55%)	24 (63%)	17 (47%)	0.67
Etiology of PH				
Idiopathic	41 (55%)	23 (61%)	18 (50%)	0.34
Chronic thromboembolic	22 (30%)	10 (26%)	12 (33%)	0.51
Lung disease	11 (15%)	5 (13%)	6 (17%)	0.63
Index ATA				
Atrial fibrillation	38 (51%)	19 (50%)	19 (53%)	0.80
Atrial tachycardia	13 (18%)	7 (18%)	6 (16%)	0.82
Typical atrial flutter	23 (31%)	12 (32%)	11 (31%)	0.93
Comorbidities				
Arterial hypertension	59 (80%)	31 (82%)	28 (78%)	0.67
Diabetes mellitus	26 (35%)	12 (32%)	14 (39%)	0.53
Coronary artery disease	14 (19%)	5 (13%)	9 (25%)	0.19
Stroke/transient ischemic attack	6 (8%)	4 (11%)	2 (6%)	0.44
Antiarrhythmic treatment				
Amiodarone	14 (19%)	8 (21%)	6 (17%)	0.66
Propafenone	3 (4%)	2 (5%)	1 (3%)	0.66
Functional status				
NYHA class I	0	0	0	1.0
NYHA class II	17 (23%)	9 (24%)	8 (22%)	0.84
NYHA class III	57 (77%)	29 (76%)	28 (78%)	0.84
NYHA class IV	0	0	0	1.0
NT-proBNP (pg/mL)	1,267 (732; 2,317)	903 (724; 1,979)	1,587 (922; 3,182)	0.11
LV ejection fraction (%)	60 (55; 63)	60 (55; 62)	60 (56; 64)	0.34
Hemodynamics				
PAMP (mmHg)	46 (38; 55)	47 (38; 54)	45 (36; 55)	0.60
PCWP (mmHg)	11 (9; 15)	12 (10; 19)	11 (9; 13)	0.18

The data represent the number of cases (percentage) or median (interquartile range).

ATA, atrial tachyarrhythmia; LV, left ventricle, NS, not significant, NT-proBNP, N-terminal pro-B-type natriuretic peptide; PAMP, pulmonary artery mean pressure, PCWP, pulmonary capillary wedge pressure; PH, pulmonary hypertension.

### 3.1 Original “short-term” follow-up

The median follow-up duration in the original trial was 13 months (IQR: 12; 18) in the limited ablation group and 14 months (IQR: 12; 21) in the extended ablation group. The primary endpoint occurred in 15 patients (42%) in the extended ablation group vs. 17 patients (45%) in the limited ablation group [hazard ratio (HR): 0.97, 95% confidence interval (CI): 0.49–2.0]. Ten patients (28%) from the extended group died, and there were 9 deaths (24%) in the limited ablation group (HR: 0.92, 95% CI: 0.36–2.3).

### 3.2 Long-term follow-up

**Figure 1:**
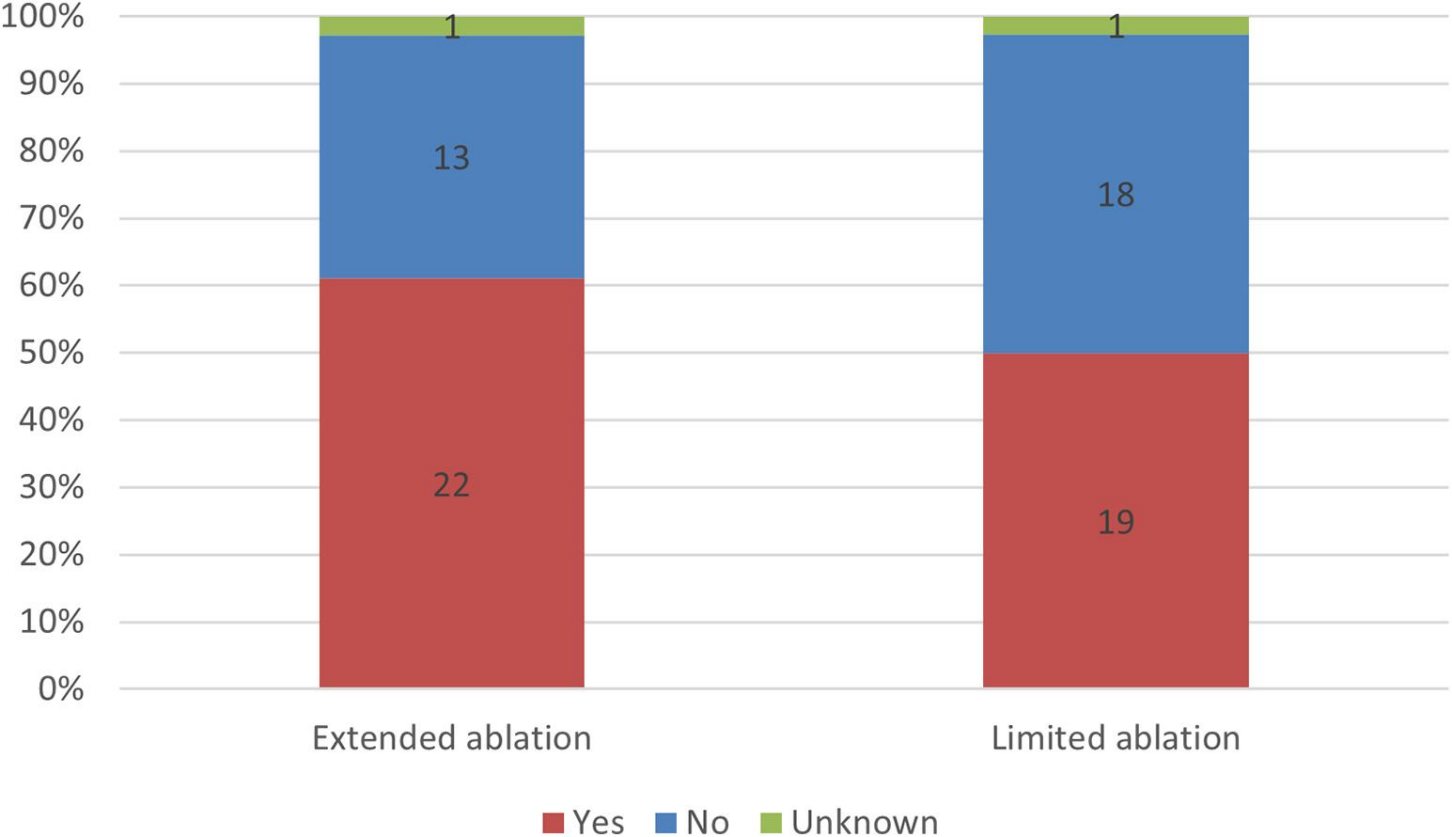
The presence of a primary endpoint (ATA recurrence) during the long-term follow-up according to study groups. Red—ATA recurrence; blue—no ATA recurrence; green—primary outcome not known.

The median duration of prolonged follow-up was 45 months (IQR: 20; 59) in the extended ablation group and 41 months (IQR: 17; 57) in the limited ablation group. During this time, the primary endpoint occurred in 22 patients (61%) after extended ablation and in 19 patients (50%) after limited ablation (HR: 1.2, 95% CI: 0.66–2.3, Figure 1**)**. Unfortunately, we were unable to assess the primary endpoint in one patient from the extended ablation group and in one from the limited ablation group.

**Figure 2:**
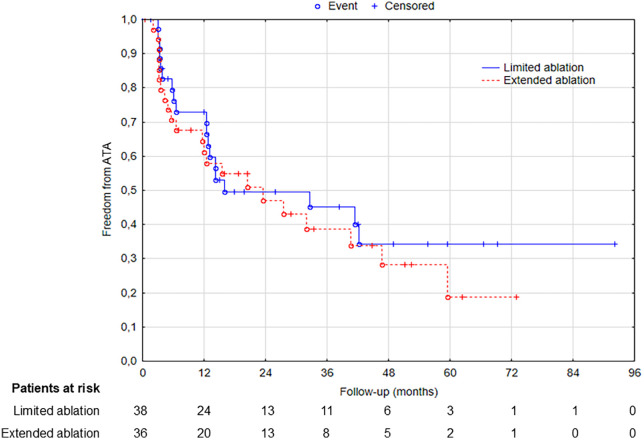
Kaplan–meier curves for ATA-free survival (primary endpoint).

Late recurrence of ATA (i.e., after completion of the original trial) was observed in 7 subjects treated with the extensive ablation strategy and in 2 patients treated with the limited ablation strategy. Figure 2 shows the event-free survival (Kaplan–Meier curves) for the ATA recurrence by study group assignment. The median time to ATA recurrence was 9 months (IQR: 3; 23) vs. 12 months (IQR: 4; 14) in the extended vs. limited ablation groups, respectively.

**Figure 3:**
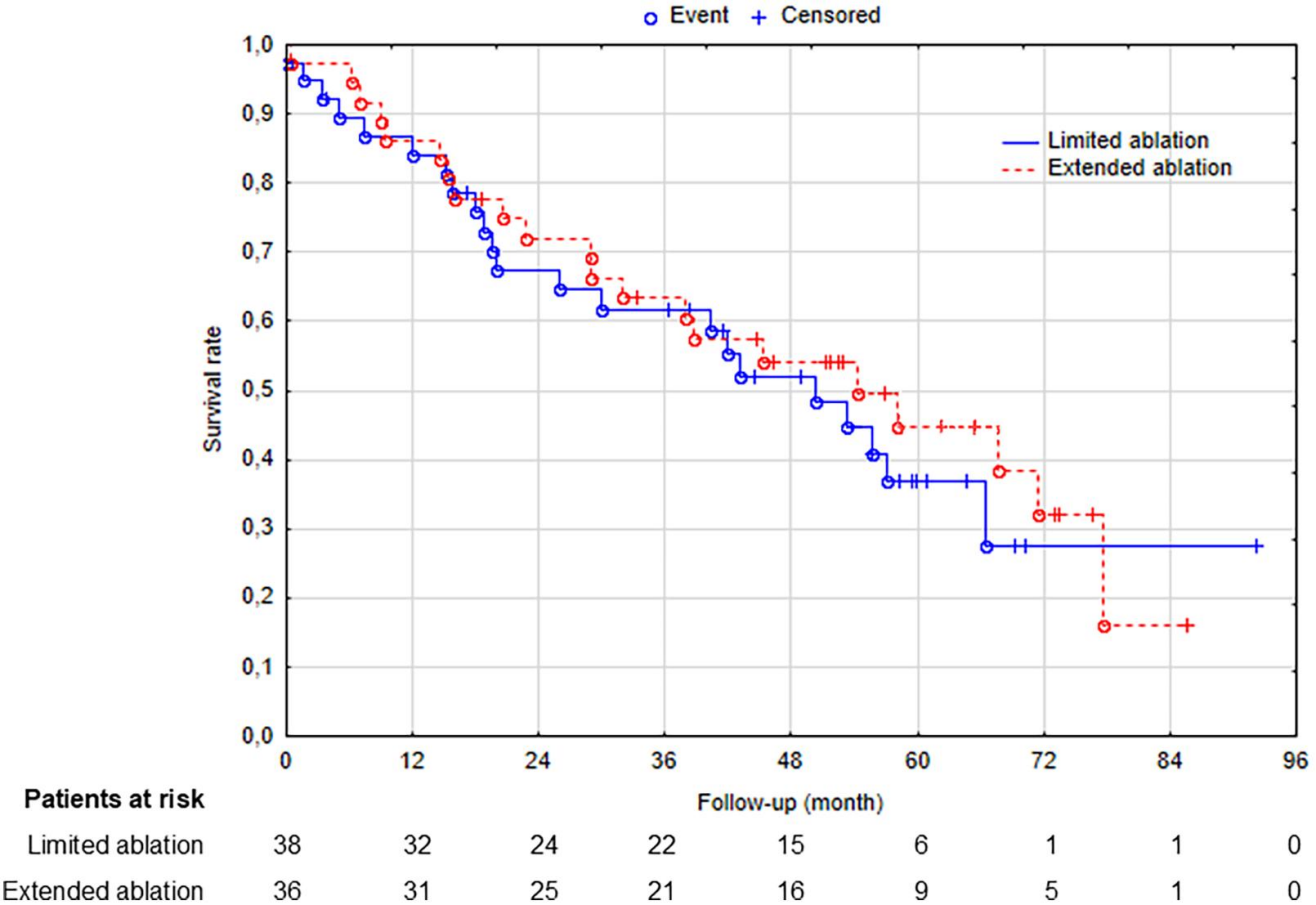
Kaplan–meier survival curves for all-cause death (secondary endpoint).

By the end of prolonged follow-up, there were 21 deaths (58%) in the extended ablation group and 22 patients (58%) died after the limited ablation (HR: 0.74, 95% CI: 0.39–1.4). The median time to death was 29 months (IQR: 15; 49) vs. 20 months (IQR: 12; 43) in the extended vs. limited ablation groups, respectively. Figure 3 shows the Kaplan–Meier curves for all-cause death by study group assignment.

Eventually, ATA recurrence was observed in 41/74 patients (55%) regardless of the original study group assignment. After the ATA recurrence, 23/41 patients (56%) died. However, 31/74 (42%) subjects maintained sinus rhythm. The mortality of this subgroup was comparable, 18/31(58%) deaths. Using a Cox proportional hazards model, ATA recurrence was not significantly associated with mortality (HR: 0.68, 95% CI: 0.36–1.26).

Overall, 23 patients were ablated for typical atrial flutter. Interestingly, we did not observe any recurrence of this arrhythmia during prolonged follow-up. However, different ATAs occurred in 6/11 (55%) patients in the extended ablation group vs. 3/12 (25%) patients treated with ablation limited to the cavo-tricuspid isthmus (*P* = 0,14). Among these patients, atrial fibrillation was documented in seven cases, while two patients were diagnosed with a different type of ATA.

## 4 Discussion

Our study shows that the recurrence rate of ATAs after CA in patients with PH is particularly high and not affected by the extend of ablation. During the long-term follow-up of almost 4 years, ATA recurred in 61% of patients after extended ablation vs. 50% in the limited ablation group. The difference between the study groups was not statistically significant. High ATA recurrence rates after CA are typically reported in PH patients compared to the general population (10–12, 14, 15).

Mortality was similarly high, at 58% in both study groups. One-year mortality for patients with pulmonary arterial hypertension (PAH) is usually reported to be 1%–21%, depending on the patient's risk profile. The three-year mortality of about 50% is then comparable to that of high-risk patients with PAH (16, 17). Unfortunately, we were unable to determine the cause of death in individual patients. We speculate, based on the available incomplete follow-up data, that most patients from our study population died of progressive heart failure caused by pulmonary hypertension. This is in line with the published data, as progressive right heart failure has been reported as the most common cause of death in PAH patients (18).

Several studies have shown that ATA in PH patients is associated with further clinical deterioration (5–7, 11, 12, 19–22). Furthermore, some studies suggested that sustained ATA was associated with increased mortality in PH (5, 12, 19, 20). However, the mortality of our patients was not affected by ATA recurrence, as it was similarly high in those who maintained sinus rhythm throughout follow-up. Unfortunately, we do not have sufficient data to distinguish between patients with recurrent paroxysmal and persistent ATA. A considerable proportion of patients were ablated for typical atrial flutter. In agreement with the results of the original trial (13), we did not observe any recurrence of the typical atrial flutter after the index ablation, even during long-term follow-up. Therefore, regardless of the study group assignment, we were able to create a durable block at the cavo-tricuspid isthmus in most patients. Alternatively, recovered gaps at the isthmus likely had unstable conduction properties, rendering the episodes of typical flutter non-sustained and short. This far more favourable result compared with previously published trials (11, 12) can be explained by the use of 3D-electroanatomical mapping and intracardiac echocardiography in the study population. Additionally, frequent use of steerable sheaths and the operator's experience may play a role (23). However, even patients after successful typical atrial flutter ablation are at high risk of developing different ATAs according to our results and earlier studies (11, 12).

The pathophysiology underlying ATA development likely differs according to the type of PH. Chronic right ventricular pressure overload and right atrial enlargement, leading to myocardial hypertrophy and electrical remodelling, are considered particularly important in patients with idiopathic PAH and chronic thromboembolic PH. In patients with PH associated with lung disease, the effects of chronic hypoxemia, hypercapnia, acidosis, inflammation, and oxidative stress may contribute to arrhythmogenesis. In contrast, distinct mechanisms related to left atrial enlargement and fibrosis predominate in patients with PH due to left heart disease (24–26). Although the original trial excluded patients with isolated post-capillary PH, it included patients with idiopathic PAH, PH associated with lung disease, and chronic thromboembolic PH. This heterogeneity in PH etiology and severity may have influenced both the efficacy of catheter ablation and long-term outcomes. However, after randomization, no statistically significant differences in PH etiology or severity were observed between the limited and extended ablation groups (Table 1).

We are aware that the presented study has several limitations. The most important of them is the absence of a prespecified protocol for the long-term follow-up. The frequency of patients' visits was driven by the clinical situation. The 12-lead ECG was a standard part of each visit, but the indication for prolonged ECG monitoring was left to the discretion of the attending physician. There is missing data as we have lost 2 patients for follow-up. Also, due to limited and/or missing data, we were unable to assess the exact type of recurrent ATA in every single patient or its impact on the patient's quality of life and functional status.

All patients in our trial underwent radiofrequency CA, as the pulsed field technology was not yet available at the time of enrolment. According to our current clinical practice, a significant proportion of patients in this trial (mainly those with atrial fibrillation) would likely be treated with pulsed field ablation under general anaesthesia. The recurrence rate of atrial fibrillation after pulsed field ablation is comparable to that of radiofrequency ablation in the general population (27, 28). However, we can only speculate how pulsed field technology would have affected the outcomes of PH patients in our trial.

Despite the limitations mentioned, we are convinced that the results of this analysis give important and novel data about ATAs management and the strategy of CA in PH patients. Our findings are based on the randomized population managed in three tertiary cardiac centres with expertise in PH and invasive electrophysiology. The number of subjects is limited and may be underpowered to prove statistically significant difference in long-term outcomes but still quite high compared to other trials focused on CA in patients with PH (29).

## 5 Conclusion

The extended radiofrequency CA did not reduce ATA recurrence in patients with PH compared with a limited ablation strategy during long-term follow-up of almost 4 years. Despite the ablation, the recurrence rate of ATAs in PH patients was particularly high. The prognosis of this patient population remains poor, and we were unable to demonstrate an improvement associated with maintenance of sinus rhythm during follow-up. According to our findings, cavo-tricuspid isthmus ablation appears to be effective in preventing recurrence of typical atrial flutter, even in patients with PH. However, as this was a secondary analysis with several limitations, the results should be considered hypothesis-generating. Further research is needed to better understand the impact of ATAs in patients with PH and to define the optimal treatment strategy for this population.

## Data Availability

The original contributions presented in the study are included in the article/Supplementary Material, further inquiries can be directed to the corresponding author.
